# Bovine Lactoferrin Counteracts Toll-Like Receptor Mediated Activation Signals in Antigen Presenting Cells

**DOI:** 10.1371/journal.pone.0022504

**Published:** 2011-07-25

**Authors:** Patrizia Puddu, Daniela Latorre, Maria Carollo, Angela Catizone, Giulia Ricci, Piera Valenti, Sandra Gessani

**Affiliations:** 1 Department of Cell Biology and Neurosciences, Istituto Superiore di Sanità, Rome, Italy; 2 Department of Anatomy, Histology, Forensic Medicine and Orthopedics, Sapienza, University of Rome, Rome, Italy; 3 Department of Experimental Medicine, Second University of Naples, Naples, Italy; 4 Department of Public Health Sciences and Infectious Diseases, Sapienza, University of Rome, Rome, Italy; Tulane University, United States of America

## Abstract

Lactoferrin (LF), a key element in mammalian immune system, plays pivotal roles in host defence against infection and excessive inflammation. Its protective effects range from direct antimicrobial activities against a large panel of microbes, including bacteria, viruses, fungi and parasites, to antinflammatory and anticancer activities. In this study, we show that monocyte-derived dendritic cells (MD-DCs) generated in the presence of bovine LF (bLF) fail to undergo activation by up-modulating CD83, co-stimulatory and major histocompatibility complex molecules, and cytokine/chemokine secretion. Moreover, these cells are weak activators of T cell proliferation and retain antigen uptake activity. Consistent with an impaired maturation, bLF-MD-DC primed T lymphocytes exhibit a functional unresponsiveness characterized by reduced expression of CD154 and impaired expression of IFN-γ and IL-2. The observed imunosuppressive effects correlate with an increased expression of molecules with negative regulatory functions (i.e. immunoglobulin-like transcript 3 and programmed death ligand 1), indoleamine 2,3-dioxygenase, and suppressor of cytokine signaling-3. Interestingly, bLF-MD-DCs produce IL-6 and exhibit constitutive signal transducer and activator of transcription 3 activation. Conversely, bLF exposure of already differentiated MD-DCs completely fails to induce IL-6, and partially inhibits Toll-like receptor (TLR) agonist-induced activation. Cell-specific differences in bLF internalization likely account for the distinct response elicited by bLF in monocytes *versus* immature DCs, providing a mechanistic base for its multiple effects. These results indicate that bLF exerts a potent anti-inflammatory activity by skewing monocyte differentiation into DCs with impaired capacity to undergo activation and to promote Th1 responses. Overall, these bLF-mediated effects may represent a strategy to block excessive DC activation upon TLR-induced inflammation, adding further evidence for a critical role of bLF in directing host immune function.

## Introduction

Lactoferrin (LF) is an 80 kDa iron-binding glycoprotein abundantly found in most biological fluids of mammals, which binds with high affinity two ferric ions per molecule [1]. It is secreted in an iron-free form from many mucosal epithelia cells, and released by neutrophils during inflammation. LF is now recognized as a key element in the host defence system [2], [3], [4]. In addition to the antimicrobial properties, both human recombinant and bovine native LFs exhibit a variety of effects on the host immune system, ranging from inhibition of inflammation to promotion of both innate and adaptive immune responses [2], [5]. Although the mechanisms underlying LF immunomodulatory properties have not been fully elucidated yet, evidence indicates that the capacity of this molecule to directly interact with antigen presenting cells (APCs), i.e. monocytes/macrophages and dendritic cells (DCs) may play a critical role. At the cellular level, LF modulates important aspects of APC biology, including migration and cell activation, whereas at the molecular level it affects expression of soluble immune mediators, such as cytokines, chemokines and other effector molecules, thus contributing to the regulation of inflammation and immunity [2], [5].

Among APCs, monocytes/macrophages and DCs are of critical importance for the maintenance of tissue homeostasis and innate response to pathogens, as well as in linking innate to adaptive immune response. Monocytes/macrophages are highly phagocytic cells which play a central role in the control of infection, either by direct intracellular killing of microorganisms or by secreting cytokines inhibiting their replication, as well as in type II inflammation and tissue repair processes [6], [7]. DCs are a heterogeneous population of cells highly specialized for antigen recognition that play a key role in the immune system by virtue of their capacity to control both immune activation and tolerance induction [8], [9]. Inflammatory mediators and especially the Toll like receptor (TLR) family of proteins have been shown to play a pivotal role in inducing the immune activation program in DCs. The activation of resting DCs is a crucial step in the initiation of adaptive immunity as it links peripheral events initiated by the encounter with pathogens to the activation and expansion of antigen specific T lymphocytes in secondary lymphoid organs [10].

However, accumulated evidence highlights the functional plasticity of DCs, which are also able to drive antigen-specific unresponsiveness or tolerance in central lymphoid organs and in the periphery, and to contribute to the expansion and differentiation of T cells that regulate or suppress other immune T cells [11].

In the present study, we report that bovine LF (bLF) acts as a potent anti-inflammatory agent on monocytes by triggering a tolerogenic-like program during their differentiation into DCs. MD-DCs generated in the presence of bLF show enhanced expression or activation of molecules characterizing tolerogenic DCs, and constitutively secrete IL-6. Consistent with their phenotypic features, T lymphocytes expanded in bLF-exposed DC/T lymphocyte co-cultures are anergic and produce little IFN-γ and IL-2. Major differences have been observed in the capacity of monocytes to internalize bLF with respect to MD-DCs thus providing a mechanistic base for the rather divergent effects of bLF, anti-inflammatory *versus* immunostimulatory, observed in monocytes and DCs, respectively.

Overall, the capacity of LF to differently interact with specific cell types of the immune system leading to both induction and suppression of the immune response adds further evidence to the pivotal role of this natural compound in the orchestration of the immune response.

## Results

### Phenotypic properties of MD-DCs generated in the presence of bLF

**Figure 1:**
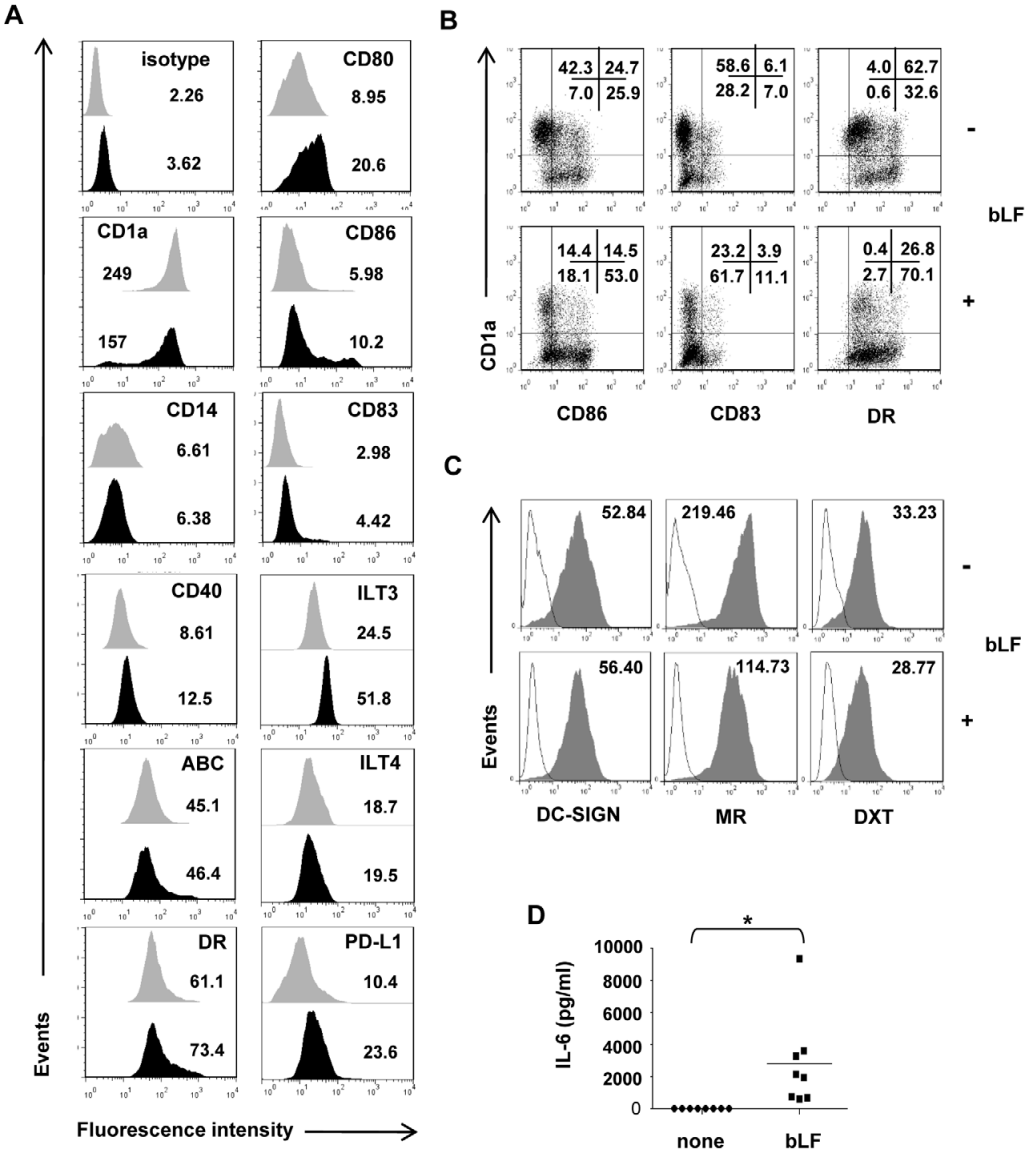
Phenotypic characterization of iMD-DCs generated in the presence of bLF. Monocytes were stimulated to differentiate into MD-DCs in the absence or in the continuous presence of 100 µg/ml of bLF. Bovine LF was added to the culture at day 0, 2 and 5 concomitantly to the addition of GM-CSF and IL-4. At day 6, cells and culture supernatants were collected. (A) Cells were stained with the indicated mAbs as described in “Materials and Methods”, and analyzed by flow cytometry. The shaded and black areas represent the expression of phenotypic markers in control and bLF-treated cells, respectively. Numbers indicate MFI of markers analyzed on cells. One representative experiment out of 4 performed is shown. (B) FACS dot plots show PE-conjugated CD1a *versus* FITC-conjugated CD86, CD83 or HLA-DR staining. Numbers indicate percentages of cells included in each quadrant. One representative experiment out of 4 performed is shown. (C) Cells were stained with specific mAbs to DC-SIGN, MR or FITC-conjugated DXT, and analyzed by flow cytometry. Open histograms represent the background staining of isotype-matched mAbs for DC-SIGN and MR, or cells incubated with DXT at 0°C. Numbers indicate MFI of DC-SIGN, MR and DXT staining. One representative experiment out of 4 is shown. (D) Six-day supernatants from bLF-treated or untreated cultures were analyzed by ELISA for IL-6 content. *p* values (n = 8, *p<0.05) were calculated for IL-6 production in cultures exposed to bLF *versus* control cells.

**Figure 2:**
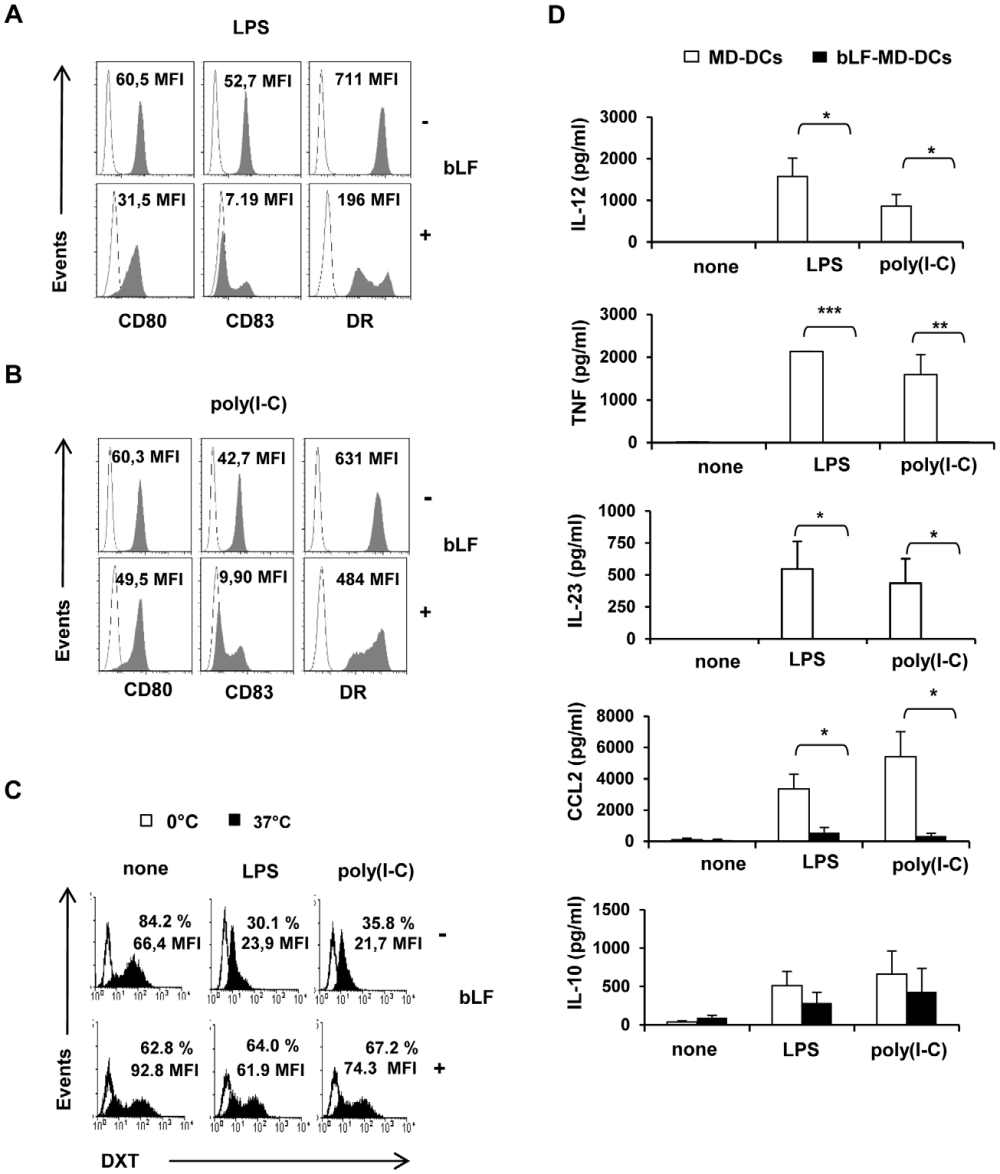
bLF-induced impairment of MD-DC maturation. Monocytes were stimulated to differentiate into iMD-DCs in the presence or in the absence of bLF as described in the legend to Figure 1. At day 5 of culture, cells were stimulated with LPS (10 ng/ml) (A) or poly(I-C) (20 µg/ml) (B). Twenty-four hours later, cells were collected and stained with mAbs to CD80, CD83 and HLA-DR. Open area represents staining with isotype Ab and shaded area phenotypic markers expression in activated control and bLF-MD-DCs. One representative experiment out of 4 is shown. (C) Control and bLF-MD-DCs were stained with DXT 24 h after LPS or poly(I-C) stimulation, and analyzed by flow cytometry. Open and black histograms represent staining with DXT at 0°C or 37°C, respectively. Numbers indicate the percentage of positive cells and MFI values. One representative experiment out of 4 is shown. (D) Cytokine and chemokine contents in supernatants of MD-DCs stimulated with LPS or poly(I-C) for 24 h. Mean ± SE of 6 to 14 independent experiments is shown. * *p*<0.05, ** *p*<0.01, *** *p*<0.001, MD-DCs *versus* bLF-MD-DCs.

The expression of a panel of surface antigens, typical of immature DCs, was analyzed in monocytes stimulated to differentiate into classic MD-DCs in the absence or in the continuous presence of bLF. As shown in **Figure 1A**, bLF did not interfere with monocyte differentiation into MD-DCs since bLF exposed cells at day 6 of culture expressed CD1a, CD80, CD86 and CD40, as well as MHC class I and II antigens, and barely detectable levels of CD83 and CD14 consistent with their differentiation into immature MD-DCs (iMD-DCs). However, a modest but reproducible increase in the expression of CD80, CD86 and HLA-DR, and to a higher extent of programmed death ligand-1 (PD-L1) and immunoglobulin-like transcript 3 (ILT3) was observed in bLF generated MD-DCs (bLF-MD-DCs) with respect to control iMD-DCs. Conversely, ILT4 was expressed at comparable levels in control and bLF-MD-DCs. Furthermore, bLF influenced the dichotomy CD1a^−^/CD1a^+^ observed in *in vitro* generated MD-DCs [12], [13]. The percentage of CD1a^+^ cells monitored in ten independent monocyte cultures substantially varied (*p*<0.05) when cells were differentiated in the presence of bLF, with a preferential generation of CD1a^−^ cells (mean 18±6%, n = 10) with respect to control cultures (mean 8±3%, n = 10). Interestingly, the majority of CD1a^−^ MD-DCs also expressed high levels of HLA-DR and CD86, and some of them were positive for the activation marker CD83 (**Figure 1B**). In keeping with their immature phenotype, bLF-MD-DCs expressed high levels of dendritic cell-specific ICAM-3 grabbing nonintegrin (DC-SIGN) and mannose receptor (MR), and exhibited a high capacity to uptake dextran (DXT) (**Figure 1C**). Likewise, bLF-MD-DCs did not secrete effector cytokines typical of activated DCs, including IL-12, TNF, IL-23, IL-10 and CCL2 (see **Figure 2C**). Interestingly, bLF-MD-DCs produced high levels of IL-6 that were not found in control cultures (**Figure 1D**).

### bLF generated MD-DCs do not undergo phenotypic and functional maturation following TLR stimulation

Next we evaluated whether bLF-MD-DCs could mature in response to TLR triggering. As shown in **Figure 2A**, up-modulation of CD80, HLA-DR and CD83, consistently observed in LPS stimulated control cultures, was markedly reduced in bLF-MD-DCs.

Structurally, LFs of different origins contain a highly basic region close to the N-terminus which binds to a variety of anionic biological molecules, including lipid A, with high affinity [14], [15]. To exclude that the observed bLF-mediated inhibitory effect on DC activation was due to the capacity of this molecule to sequester LPS thus neutralizing its biological activity, we tested the effect of bLF on the maturation induced by other TLR agonists such as the TLR3 ligand poly(I-C). As shown in **Figure 2B**, poly(I-C) induced phenotypic changes were strongly reduced in bLF-MD-DCs as compared to control cultures. Likewise, CD83 up-modulation was also inhibited in bLF-MD-DCs stimulated with the imidazoquinoline compound resiquimod (R848) with respect to control cultures (**Figure S1, panel A**). These results provide evidence that inhibition of MD-DC maturation is not merely related to bLF capacity to bind LPS but likely relies on direct effects of this molecule on its target cells. In keeping with the lack of phenotypic changes indicative of DC maturation, bLF-MD-DCs retained a high capacity to uptake DXT upon activation with both LPS and poly(I-C), consistent with an immature phenotype (**Figure 2C**). As expected, the high endocytic capacity exhibited by control MD-DCs at the immature state was markedly down-modulated upon maturation induction with LPS or poly(I-C). Likewise, bLF-MD-DCs treated with LPS and poly(I-C) failed to produce or produced remarkably less IL-12 (*n* = 6, *p*<0.05 for both stimuli), TNF (*n* = 11, *p*<0.001 and *p*<0.002 for LPS and poly(I-C), respectively), IL-23 (*n* = 14, *p*<0.05 for both stimuli) and CCL2 (*n* = 7, *p*<0.05 for both stimuli) than control MD-DCs stimulated under the same conditions (**Figure 2D**). Likewise, IL-12 and TNF up-modulation was markedly inhibited in bLF-MD-DCs stimulated with R848 with respect to control cultures (**Figure S1**, **panels B and C**). In contrast, no significant differences were observed in the production of IL-10 that was up-modulated at a comparable extent in both control MD-DC and bLF-MD-DC cultures stimulated with LPS or poly(I-C) (*n* = 7, *p* = 0.298 and *p* = 0.228, respectively) (**Figure 2D**). These results exclude that the bLF-mediated inhibitory effect on cytokines/chemokines relies on hyper-production of IL-10, a cytokine well-known as a negative regulator of IL-12 and other cytokines [16].

### bLF differently interacts with DC precursor cells and differentiated immature DCs

Previous studies demonstrated that exposure of iMD-DCs to human recombinant LF (Talactoferrin) results in their functional activation and promotes Th1 responses [17], [18]. From the results achieved to this point it was obvious that stimulation with bLF during DC generation suppresses the development of functional DCs suggesting that this molecule could differently interact with differentiated DCs and their monocyte precursors. In fact, in our study, cells have been treated with bLF at different time of culture, immediately after monocytes isolation and every three days until sample processing, thus allowing bLF to exert differential effects on the two different cell targets.

**Figure 3:**
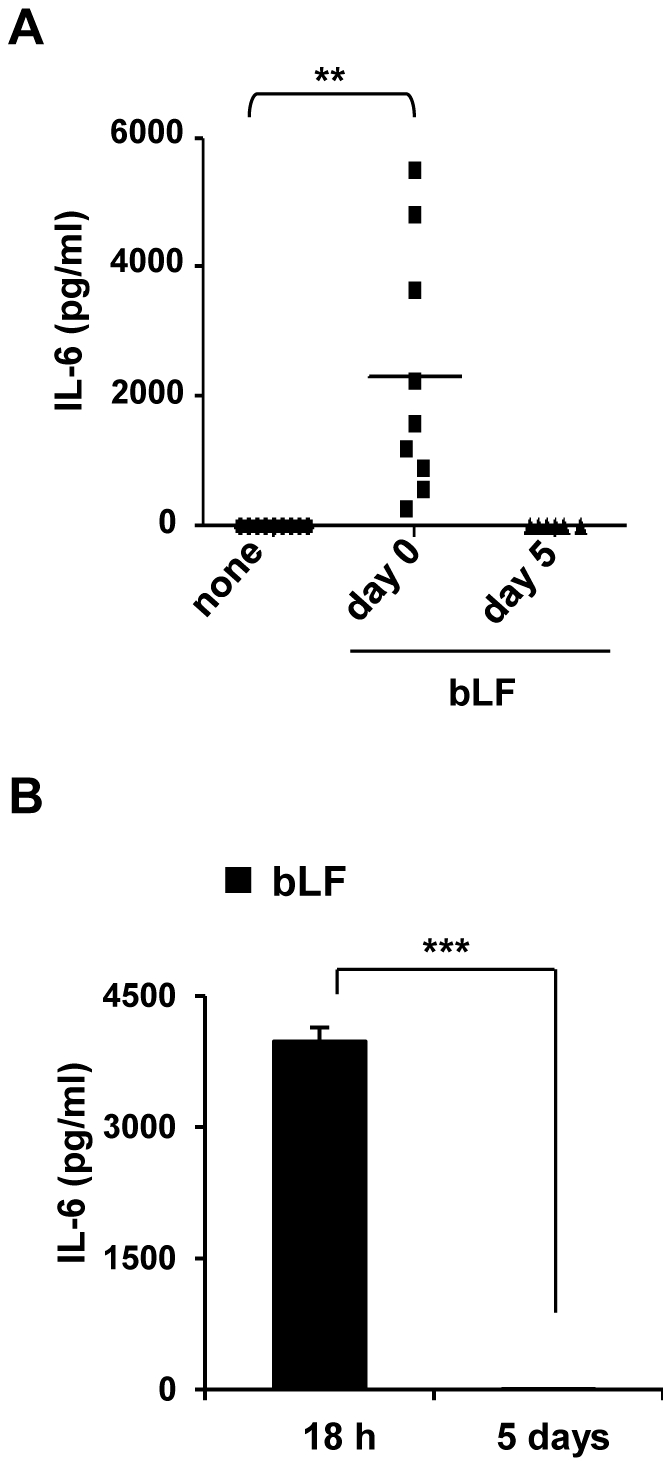
bLF induces an early and transient IL-6 production in DC precursor cells but not in iMD-DCs. Monocytes were treated with bLF, in the presence of GM-CSF and IL-4. (A) bLF was added once to cultures soon after seeding (day 0) or at day 5. Controls were left untreated (none). At day 6, supernatants were assessed for IL-6 content. The results of independent experiments are shown (bLF day 0 *versus* untreated, n = 13, **p<0.01). (B) Cells were treated with bLF for 18 h, then the culture medium was replaced with fresh medium containing GM-CSF and IL-4, and cells cultured for additional 5 days. IL-6 secreted in the 18 h and 5 day conditioned medium was titrated by ELISA. Mean ± SE of 5 independent experiments is shown (*** *p*<0.001).

To explore this hypothesis, we assessed the effect of a single treatment with bLF of freshly isolated monocytes (day 0) or iMD-DCs (day 5) on the 24 h production of IL-6. As shown in **Figure 3A**, high levels of IL-6 were found in monocyte cultures treated with bLF (n = 13, p<0.01) while this cytokine was not secreted when bLF was added to iMD-DCs (n = 6, p = 0.363). Conversely, other cytokines such as IL-10 and IL-12 were not expressed following bLF treatment in both freshly isolated monocytes and iMD-DCs (data not shown). IL-6 production entirely occurred within the first 18 h after cell seeding. In fact, when the culture medium of bLf stimulated monocytes was replaced with fresh medium 18 h post stimulation and cells were cultured for additional 5 days, no IL-6 was detected in the supernatant at the end of cultures (**Figure 3B**).

### bLF is internalized by monocytes but not immature DCs and accumulates into the nucleus

**Figure 4:**
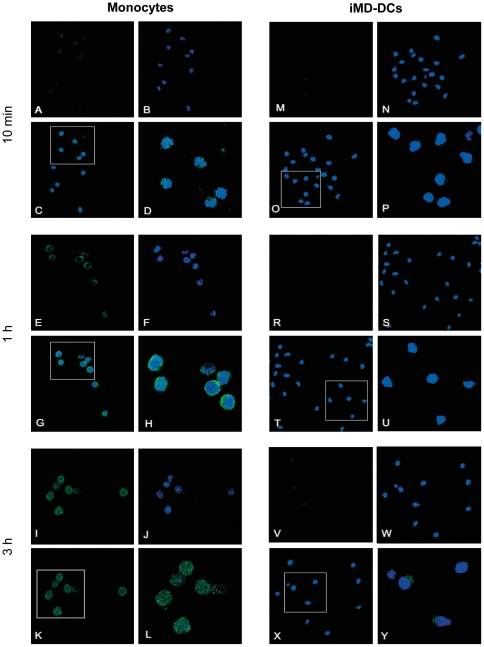
Differential bLF internalization in monocytes and iMD-DCs. CLSM images of cell-associated bLF in monocytes and MD-DCs treated once with bLF (100 µg/ml) soon after seeding or after 6 days of culture, respectively, at the indicated time-points after treatment. Images taken at level of the nuclear section of one representative experiment out of 3 performed are shown. Single green and blue fluorescences represent bLF (panels A, E, I, M, R, V) and nuclei (panels B, F, J, N, S, W), respectively. Panels C, G, K, O, T, X, and their respective 2× magnification (panels D, H, L, P, U. Y) show merged green and blue fluorescence.

**Figure 5:**
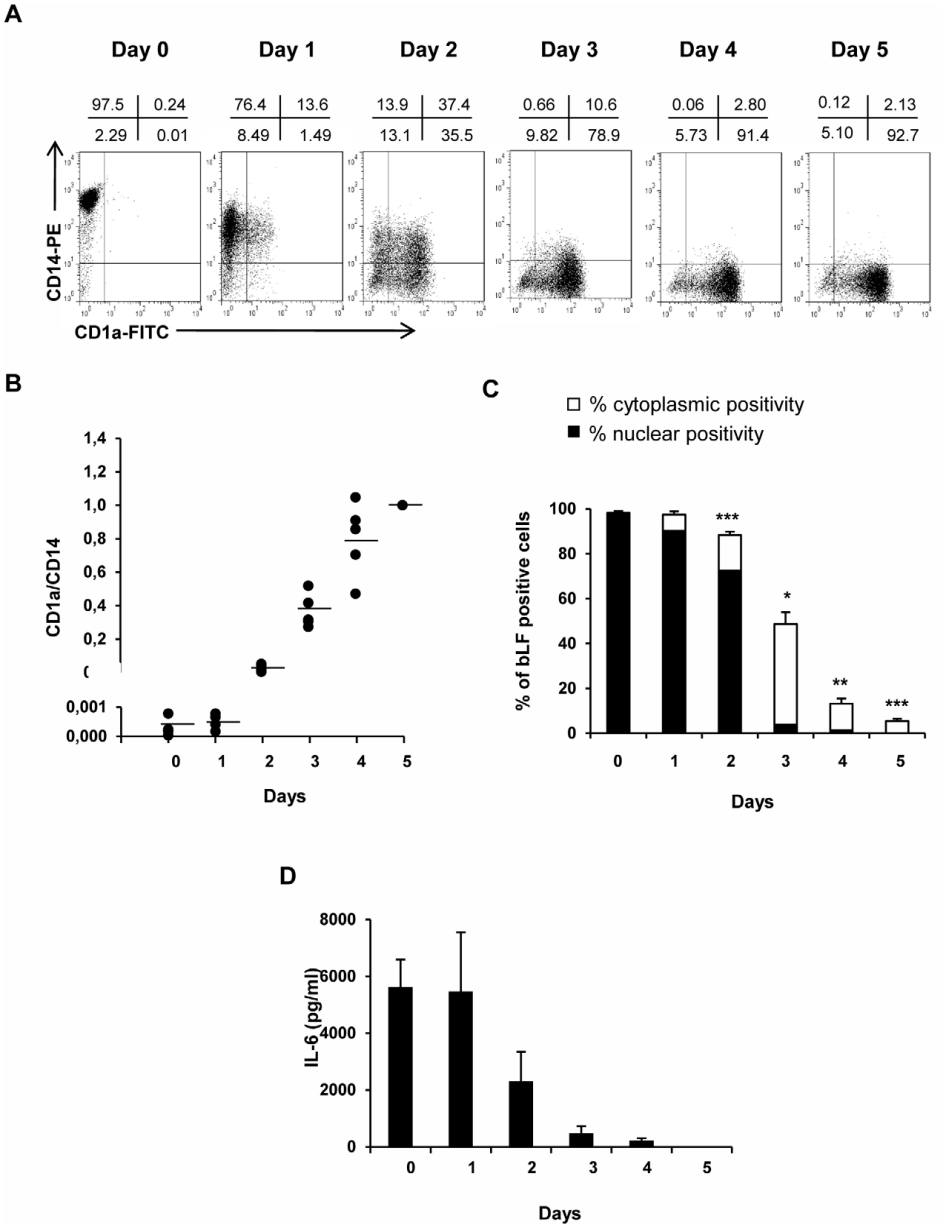
bLF internalization during monocyte to MD-DC differentiation progression. Monocytes were stimulated to differenziate into MD-DCs in the presence of GM-CSF and IL-4. (A) Flow cytometric analysis showing cell-surface phenotype during MD-DC differentiation. Monocytes and DCs can be distinguished by their CD14 and CD1a expression profiles. Data from one representative donor out of 5 are shown. (B) Quantification of CD1a/CD14 expression ratios in 5 independent donors. Ratios were calculated using MFI of CD1a/MFI of CD14 and normalized to the day 5 ratio of each donor, which was set at 1.0. (C) Monocytes were exposed to bLF soon after seeding (day 0) or at day 1, 2, 3, 4 or 5 of culture. Three hours later, bLF internalization was assessed by CLSM. Figure shows the mean ± SE of bLF positive cells in 3 independent experiments. The percentage of positive cells was calculated by analyzing at least 350 cells for each experimental point. The nuclear or cytoplasmatic positivity is also indicated. *p* values were calculated comparing the percentage of bLF positive cells at day 1, 2, 3, 4, and 5 *versus* day 0. * *p*<0.05, ** *p*<0.01, *** *p*<0.001. (D) At each time points, IL-6 produced after a 18 h treatment with bLF was measured by ELISA. Mean ± SE of 3 independent experiments is shown.

To explore the possibility that a different interaction of bLF with monocytes and iMD-DCs underlies the distinct effects mediated by this molecule, bLF uptake and internalization was investigated by confocal microscopy in freshly isolated monocytes and iMD-DCs. Time-course experiments revealed that bLF was rapidly internalized in freshly isolated monocytes, and its sub-cellular distribution was dependent on the time point examined. As shown in **Figure 4**, bLF was distributed in spotted dots in the cytoplasm of monocytes already after 10 minutes of treatment (panels A, C, D), accumulated in the perinuclear area after 1 h (panels E, G, H), and entered the nucleus at 3 h of treatment, as demonstrated by merged green and blue fluorescence (panels K and L). Conversely, iMD-DCs failed to internalize bLF and very few cells exhibiting some bLF cytoplasmic staining were detected at the later time point (panels V, X, Y). To precisely define the differentiation stage in which changes in the capability of individual cells to respond to bLF occur, progression of monocyte to MD-DC differentiation was monitored in parallel with bLF internalization and IL-6 secretion. To assess and quantify progression, percentage of CD14^+^ and CD1a^+^ cells, and CD1a/CD14 median fluorescence intensity (MFI) ratio were used as qualitative and quantitative measure of MD-DC differentiation, respectively. As shown in **Figure 5A**, monocyte to MD-DC differentiation progresses through intermediate stages reflected by specific up-modulation of CD1a and down-regulation of CD14. Day 0 monocytes were essentially all CD1a^−^ and CD14^+^. At day 1, and more markedly at day 2, cells began to express CD1a while decreasing CD14 expression. By day 3, most cells have down-regulated CD14 and acquired the expression of CD1a. By day 4, the majority of cells were fully differentiated CD1a^+^/CD14^−^ MD-DCs. Likewise, CD1a/CD14 MFI ratio steadily increased during differentiation and showed clustering of the numerical values at each stages of differentiation (**Figure 5B**), thus providing a reliable indicator of differentiation progression. According to these metrics, a full MD-DC phenotype is acquired between day 3 and 4 of culture in the presence of GM-CSF and IL-4. Concomitant analysis of bLF uptake and internalization revealed that, at day 0 and 1 of culture, most cells internalize bLF which localizes into the nucleus (**Figure 5C**). Reduction in the percentage of cells internalizing bLF was already observed at day 2, increased at day 3 with only half of the cells positively stained for bLF, which mostly localized into the cytoplasm. The capacity to internalize bLF further decreased with differentiation progression and, at day 4 and 5 of culture, most cells did not exhibit any intracellular bLF. In keeping with these results, reduction in IL-6 secretion was already observed at day 2 of culture, while this cytokine was no longer produced in response to bLF in more differentiated cells at day 4 and 5 of culture (**Figure 5D**). These results suggest an intimate relationship between differentiation progression and capacity to internalize bLF within the nucleus. Furthermore, bLF nuclear internalization appears to be an important requisite for bLF-induced IL-6 expression.

### Role of CD14 and its co-receptors, TLR2 and TLR4, in bLF induced IL-6 production

**Figure 6:**
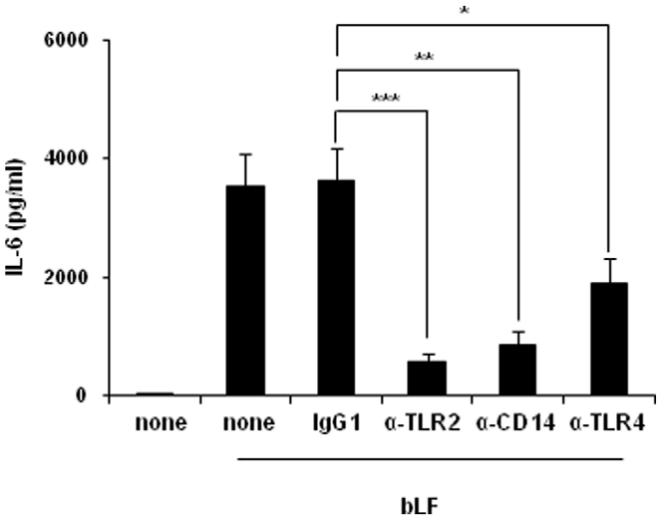
Role of CD14, TLR2 and TLR4 in bLF induced IL-6 production. Monocytes were pre-treated for 30 minutes with anti-CD14, anti-TLR2, anti-TLR4 or isotype Ab prior to bLF addition and then cultured for 6 days. Figure shows the mean ± SE of 4 independent experiments. Significance, calculated from raw data of 7 independent donors, has been evaluated comparing cells treated with bLF in the presence of isotype Ab to cells treated in the presence of Ab against TLR2, TLR4 or CD14 (isotype *versus* TLR2, n = 8, ***p<0.001; isotype *versus* CD14, n = 7, **p<0.01; isotype *versus* TLR4, n = 8, *p<0.05).

LFs bind a variety of cell determinants with different grade of specificity, including molecules involved in pathogen recognition [1], [19], [20], [21]. Experiments were then designed to define the role of CD14, TLR2 and TLR4, in bLF entry and IL-6 induction. Although blocking each of these receptors failed to affect bLF internalization in monocytes (data not shown), blocking CD14 (n = 7, *p*<0.01) and TLR2 (n = 8, *p*<0.001) with specific antibodies (Abs) strongly reduced the capacity of bLF to induce IL-6 (**Figure 6**). However, only a partial but significant reduction of IL-6 secretion was achieved when Abs against TLR4 (n = 7, *p*<0.05) were added to the cultures. Conversely, isotype control Abs did not show any effect. These results further support the hypothesis that, through CD14, bLF could preferentially target monocytes in the initial phases of DC differentiation.

### Role of IL-6 in bLF-induced inhibition of MD-DC activation

**Figure 7:**
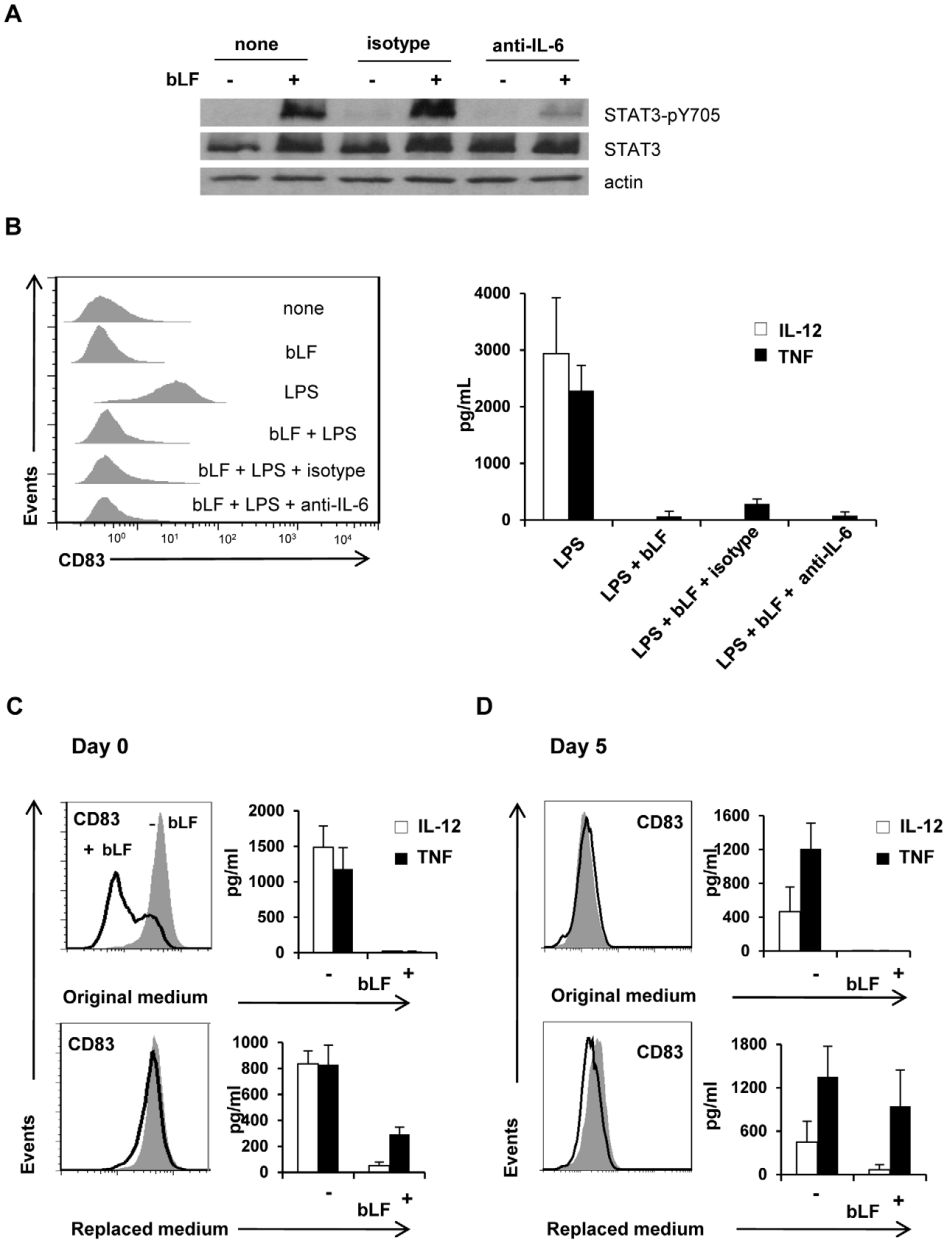
bLF-mediated effects in monocytes and iMD-DCs. (A) Monocytes were plated in complete medium containing GM-CSF and IL-4. Soon after seeding, cells were pre-treated for 30 minutes with neutralizing anti-IL-6 or isotype mAbs (5 µg/ml) or left untreated, then exposed to bLF. Treatments were repeated at day 3 and 6 of culture, and proteins were extracted 6 h after the last treatment. Immunoblotting analysis for the indicated proteins in control and bLF-exposed iMD-DCs. Samples containing 20 µg of lysate in Laemmli buffer were separated by 10% SDS-PAGE. One representative experiment out of 6 is shown. (B) Monocytes were cultured as indicated in panel A. Twenty-four h after last treatment, cells were stained with anti-CD83 or isotype mAbs and supernatants assessed for cytokine content. One representative experiment out of 4 is shown for CD83 expression. IL-12 and TNF expression is shown as mean ± SE of 3 independent experiments. (C–D) Soon after seeding or at day 5 of culture, cells were treated with bLF for 18 h, then medium was or not replaced with fresh medium (replaced medium and original medium, respectively). At day 5 cells were stimulated with LPS for 24 h. The expression of CD83, IL-12 and TNF in MD-DCs, cultured without or in the continuous presence of bLF (original medium) or exposed to bLF for 18 h (replaced medium) was assessed by FACS analysis and ELISA, respectively. Open and dashed areas represent CD83 expression in cells exposed or not to bLF, respectively. One representative experiment out of 3 performed is shown.

Previous studies demonstrated that IL-6 plays a major role in maintaining DCs at an immature state both *in vivo* and *in vitro* [22]. Importantly, signal transducer and activator of transcription 3 (STAT3) activation by IL-6 is required for the IL-6 mediated suppression of DC maturation *in vivo* [23]. Furthermore, STAT3 activation has been linked to the induction of DCs with a tolerogenic phenotype [24], [25], [26]. To address the issue of whether IL-6/STAT3 signalling could play a role in the inhibitory effect of bLF on DC activation, cell lysates prepared from control iMD-DCs and bLF-MD-DCs were analyzed for the presence of tyrosine phosphorylated STAT3. As shown in **Figure 7A**, while phosphorylated STAT3 was not detected in control MD-DCs, bLF-MD-DCs exhibited high levels of the phosphorylated form. STAT3 activation strongly depended on IL-6 since blocking the biological activity of this cytokine with specific neutralizing Abs markedly reduced the levels of phosphorylated STAT3 (**Figure 6A**). In contrast, STAT3 activation levels did not change in bLF-MD-DC cultures treated with the isotype control Ab (**Figure 6A**). Despite the fact that STAT3 activation strongly relies on IL-6, blocking the biological activity of this cytokine did not rescue the capacity of bLF-MD-DCs to undergo maturation as assessed by CD83 expression, TNF and IL-12 secretion (**Figure 7B**). However, replacement of culture medium 18 h after bLF addition, thus depleting bLF-induced soluble factors and residual bLF, partially restored the capacity of bLF-MD-DCs to respond to LPS as assessed by restoration of LPS-induced CD83 up-modulation, and partial rescue of TNF, but not IL-12 production (**Figure 7C**). Conversely, bLF addition to day 5 iMD-DCs did not block LPS-induced CD83 up-modulation while completely abrogated IL-12 and TNF secretion when the culture medium was not replaced. As expected, replacement of culture medium 18 h after bLF addition did not interfere with MD-DC capacity to up-modulate CD83 and secrete TNF in response to LPS. Conversely, IL-12 production was not rescued even after medium replacement. Thus, bLF interaction with monocytes may affect very early stages of their differentiation into DCs that, at least under certain circumstances, translate into permanent changes of activation related parameters. These changes, although not apparently involving IL-6, may at least in part rely on soluble factors released upon bLF addition.

### bLF generated MD-DCs exhibit an impaired capacity to induce T cell activation

**Figure 8:**
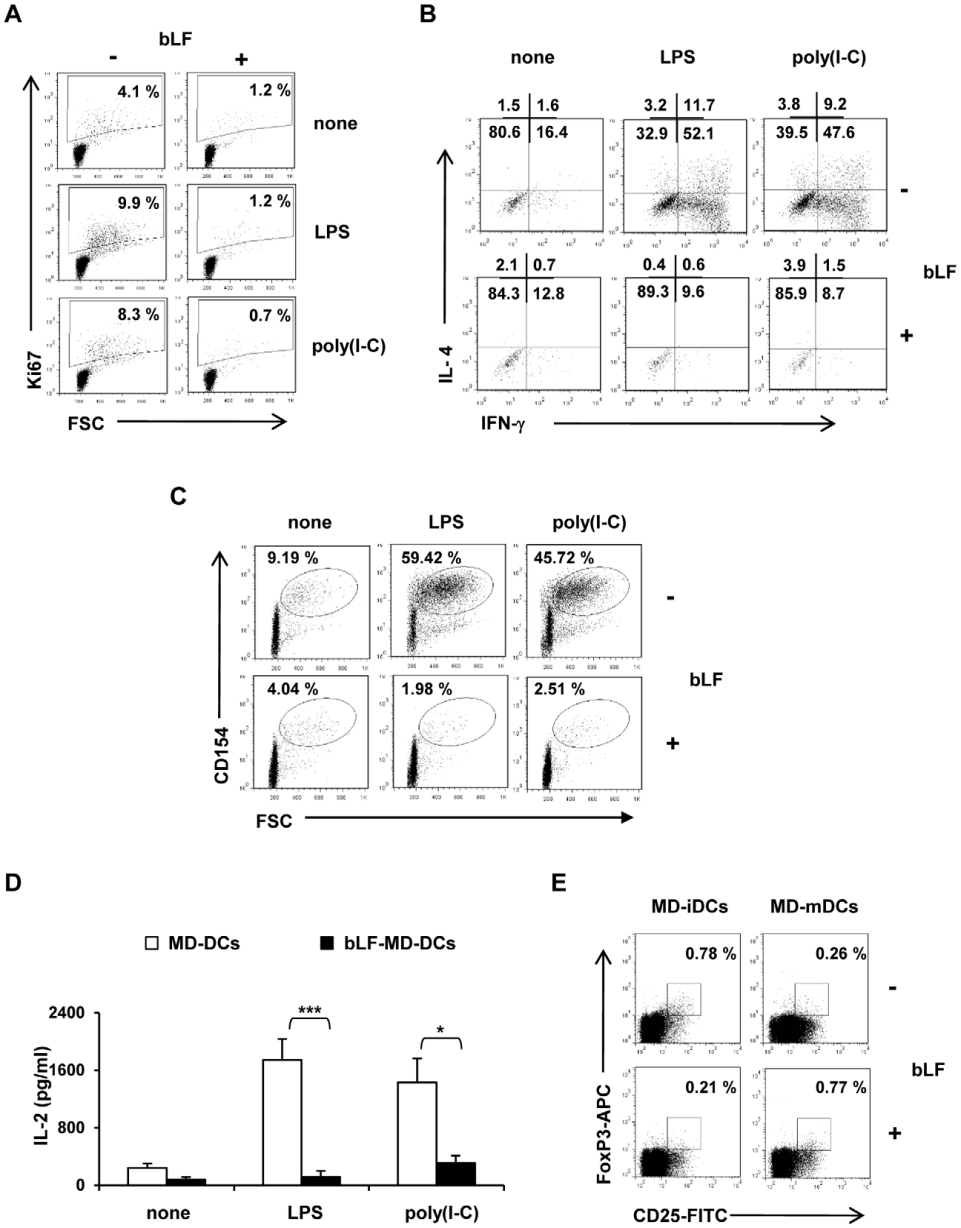
bLF inhibition of DC-mediated T cell responses. Monocytes were cultured as described in the legend to Figure 1 then stimulated with LPS and poly(I-C) for 24 h. (A) At day 6, MD-DCs were collected and co-cultured with allogenic PBL at ratio DC/PBL of 1∶100. At day 9, PBL were intracellularly stained with FITC-conjugated mAb against Ki67. FACS analysis was performed on lymphocyte population according to FSC/SSC parameters. Percentage of proliferating T lymphocytes is shown. 20.000 events were acquired per sample. (B) and (C) At day 6, DCs were co-cultured with allogenic CD4^+^ naive T cells at ratio DC/T of 1∶10 for 5 days, then stimulated with PMA (50 ng/ml) and ionomycin (1 µg/ml) then intracellularly stained with mAb to polarizing cytokines and the activation marker CD154. (B) Expression of IFN-γ and IL-4 in CD4^+^ T cells. FACS analysis was performed by gating in the region of activated lymphocytes. Numbers indicate the percentage of lymphocytes included in each quadrant. (C) Expression of CD154. A minimum of 40.000 events were acquired per sample. (D) At day 6, cells were co-cultured with allogenic CD4^+^ naive T cells at ratio DC/T of 1∶10 for 7–10 days. Supernatants were analyzed by ELISA for IL-2 content. IL-2 production is shown as mean ± SE (LPS, n = 7; poly(I-C), n = 5). Significance was evaluated comparing MD-DCs to bLF-MD-DCs co-cultures. * *p*<0.05 and *** *p*<0.001, for poly(I-C) and LPS, respectively. (E) FACS analysis of intracellular FoxP3 protein at the single cell level. CD4^+^ naive T cells were primed with control or LPS-activated MD-DCs or bLF-MD-DCs and further re-stimulated with DCs of the same donor before staining with Abs and FACS analysis. A representative FACS profile is shown as dot plots of FITC-CD25 *versus* APC-FoxP3. The quadrant gates were set according to the negative isotype control Abs in the respective experimental conditions. Numbers indicating the percentage of cells included in each quadrant are shown.

Cells generated in the presence of bLF were further characterized for their functional properties. The capacity to induce T cell activation was examined in allogenic mixed lymphocyte reactions (MLRs). After stimulation with TLR agonists, bLF-MD-DCs turned out to be weak activators of T cell proliferation, as demonstrated by the drastically reduced number of lymphocytes expressing Ki67, an intracellular antigen associated with DNA replication, with respect to control activated cells (**Figure 8A**). In keeping with these results, bLF-MD-DCs failed to prime naïve allogenic CD4^+^ T lymphocytes towards the expected Th1 polarization both in terms of percentage of IFN-γ expressing cells and the MFI for the IFN-γ positive cells (**Figure 8B**). Accordingly, IFN-γ production was not detected in the supernatant of bLF-MD-DC-T lymphocyte co-cultures with respect to control activated MD-DCs (data not shown). However, bLF-mediated impairment in IFN-γ production did not favour the expression of IL-4, since the low frequency of IL-4 producing cells was comparable in all experimental conditions (**Figure 8B**). Likewise, no IL-10 secretion was detected in the co-culture medium of both bLF exposed and control cultures (<32.5 pg/ml for each experimental point; n = 5, mean ± SE). A deeper characterization of T lymphocytes in MLRs unravelled a very low intracellular expression of the T lymphocyte activation marker CD154, comparable to that observed in iMD-DCs, in CD4^+^ T cells co-cultured with bLF-MD-DCs activated with TLR agonists with respect to T lymphocytes primed by activated control MD-DCs (**Figure 8C**). In keeping with the functional unresponsiveness of bLF-MD-DC primed T lymphocytes, a marked reduction in IL-2 content was found in LPS activated bLF-MD-DC/T lymphocyte co-culture supernatants with respect to those collected from control MD-DC/T lymphocyte co-cultures (**Figure 8D**). To exclude the possibility that the hyporesponsiveness induced by bLF-MD-DCs could be due to the preferential expansion of naturally occurring T regulatory cells, we characterized the phenotype of expanded CD4^+^ T cells. However, no major differences were observed in the percentage of FoxP3^+^/CD25^high^ T cells in MLRs with MD-DCs generated or not in the presence of bLF (**Figure 8E**).

### bLF promotes SOCS-3 and IDO expression

**Figure 9:**
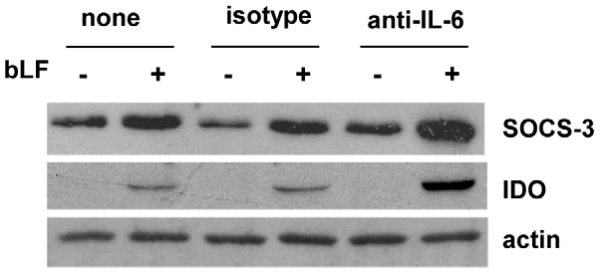
Expression of SOCS3 and IDO in bLF-MD-DCs. Cells were treated as described in the legend to Figure 6A. Immunoblotting analysis for the indicated proteins in control and bLF-exposed iMD-DCs is shown. Samples were resolved in 10% SDS-PAGE gels. One representative experiment out of 5 is shown.

Enhanced suppressor of cytokine signaling-3 (SOCS-3) expression in murine DCs has been reported to block the IL-12/IL-23 signaling in these cells and to drive them toward a tolerogenic phenotype promoting Th2 responses *in vitro* and *in vivo* [27]. Likewise, indoleamine 2,3-dioxygenase (IDO) activity in DCs has been suggested to impair T cell responses by altering the microenvironment at the DC/T cell interface [28]. Therefore, to further elucidate the mechanisms by which bLF stimulation of monocytes generates DCs with an impaired capacity to undergo activation and immunosuppressive potential, SOCS-3 and IDO expression was examined in bLF-MD-DCs. As shown in **Figure 9**, SOCS-3 expression was detected in control immature MD-DCs. However, when MD-DCs were generated in the presence of bLF a clear-cut up-modulation of SOCS-3 was observed. Conversely, despite the fact that IDO expression was not detected in control iMD-DCs, a marked induction of the expression of this enzyme was observed in bLF-MD-DCs. Interestingly, although IL-6 has been reported to up-modulate SOCS-3 in APCs [29] blocking its biological activity does not inhibit, but rather up-modulate, SOCS-3 expression. Likewise, IDO expression was up-modulated in bLF-MD-DC cultures in which IL-6 activity has been suppressed (**Figure 9**).

## Discussion

In this study, we report that human monocytes differentiated into DCs in the continuous presence of bLF exhibit most of the features of immature DCs but, upon TLR stimulation, do not acquire the phenotypic and functional properties of mature DCs as assessed by impaired up-modulation of activation related molecules and cytokines, and retention of high endocytic activity. Accordingly, bLF-MD-DCs exhibit a reduced capacity to promote the expansion of IFN-γ producing Th1 cells. However, bLF neither favours the expression of IL-4 nor modulates IL-10 production, suggesting that this molecule might control the extent of Th1 polarization rather than *per se* promoting a shift towards Th2 responses. Phenotypic and functional characterization of T lymphocytes primed by TLR agonist activated bLF-MD-DCs revealed a less activated phenotype, as assessed by a reduced expression of CD154, and a markedly diminished ability to produce IFN-γ and IL-2, suggesting a state of reduced function, namely anergy [30]. In keeping with this assumption, functional unresponsiveness of T lymphocytes has been characterized as a profound inability of CD4^+^ T cells to produce IL-2 *in vitro* [31]. Moreover, Andersen and co-workers showed that DCs generated in tolerogenic environments are capable to induce anergy in memory T cells and to skew cytokine polarization toward low IFN-γ/high IL-10 profile of naïve T cells [32]. Interestingly, anergy induction in memory T cells does not rely on the expansion of CD25^high^ T regulatory cells, and is partially reversed by IL-2. Conversely, the addition of exogenous IL-12 during DC/T cell priming preventes anergy induction in memory T cells and cytokine polarization in naïve T cells. Accordingly, we were unable to detect a preferential expansion of CD4^+^/CD25^high^ T regulatory cells in T cell cultures primed by bLF-MD-DCs.

Previous studies reported that TLR stimulation during the period of DC generation interferes with and deviates DC differentiation [33], [34], [35], [36]. In this regard, it could be argued that minimal amounts of LPS bound to bLF might have a role in the generation of tolerogenic-like DCs we described. However, experimental evidence argues against this conclusion. Specifically, we observed that blocking LPS activity by polymixin B does not completely abrogate bLF capacity to induce IL-6 (data not shown). In addition, we clearly demonstrated that the maturation arrest of bLF-MD-DCs does not merely depend on the capacity of this molecule to sequester LPS [21], as similar results have been achieved when poly(I-C) or R848 were used. Finally, bLF-MD-DCs do not show the differentiation block described for the MD-DCs generated in the presence of low amounts of LPS [33], [35]. However, our results indicate that bLF has the capacity to modulate the CD1a^+^/CD1a^−^ ratio since a modest but significant reduction in the number of CD1a^+^ DCs has been detected in the presence of bLF. Monocytes can give rise to two populations of myeloid DCs differing in CD1a expression [12], [13]. Interestingly, activated CD1a^−^ MD-DCs produce low levels of IL-12 but up-modulate IL-10 secretion, exhibit a scarce capability to induce IFN-γ production and naïve CD4^+^ Th1 polarization [12], [13], and can direct differentiation of Th0/Th2 cells [37]. Although a detailed characterization of the functional properties of bLF-induced CD1a^−^ cells has not been performed, the capacity of bLF to modulate the CD1a^+^/CD1a^−^ ratio suggests a role for this compound in the *in vivo*-relevant pathway of monocyte differentiation into DCs.

DCs have a pivotal role in both the priming of adaptive immune responses and the induction of self-tolerance. This latter function is mediated by specialized subsets of DCs, named tolerogenic DC, as well as by DC activated or differentiated in the presence of specific biological or chemical agents [11]. They all share the ability to negatively regulate T cell responses, yet their phenotypes, cytokine profiles and thus their mode of action are divergent [11]. In this regard, it has been reported that expression of PD-L1, mainly described as a negative regulatory molecule, is linked with the ability of DCs to induce tolerance [38], [39]. Likewise, the inhibitory receptor ILT3 has been shown to negatively regulate APC activation [40]. Interestingly, both molecules are up-modulated in bLF-MD-DCs suggesting that they may play a role in the negative regulation of T cell function.

Among the factors described to drive the generation of tolerogenic DCs is IL-6 [41]. This cytokine is now considered as an important mediator of the immune response especially by directly acting on CD4^+^ T cells and determining their effector functions [41], [42]. Furthermore, this cytokine promotes monocyte differentiation into tolerogenic DCs unable to produce TNF and IP-10, to induce allogenic T cell proliferation, and to express CCR7 [22]. Likewise, circulating DCs isolated from multiple myeloma patients exhibit an impaired capacity for T cell stimulation that is partly caused by IL-6-mediated inhibition of DC development [43]. However, it remains an open question how IL-6 orchestrates all these different functions but the presence of other factors will probably be a likely explanation. Studies performed in mouse models have enlighten a major role for IL-6/STAT3 signalling pathway in maintaining tolerance *in vivo* [23], [44]. In our study, we show that bLF exposed monocytes transiently produce high amounts of IL-6 soon after treatment, and this cytokine is responsible for the hyper-activation of STAT3 observed in these cells. However, blocking the biological activity of this cytokine neither rescues LPS-induced up-modulation of CD83, TNF and IL-12 nor inhibits, but rather up-modulates, the expression of tolerance related molecules, hyper-expressed in bLF-MD-DCs, such as SOCS-3 [27] and IDO [28]. Our results that IL-6 blocking is not sufficient to counteract the tolerogenic-like phenotype of bLF-MD-DCs argue against a role of this cytokine in the bLF-mediated effects. However, the observation that bLF induces IL-6 secretion and downstream STAT3 activation in monocytes but not in MD-DCs that conversely respond to bLF by up-modulating their immunostimulatory potential [17], [18], suggests that other not yet identified aspects of DC functional activation could rely on IL-6.

In this study, we report for the first time that bLF is rapidly internalized by freshly isolated monocytes, but not iMD-DCs, and reaches the nucleus. Interestingly, an intimate relationship between differentiation progression and capacity of bLF to reach the nucleus was also found. The nuclear localization of LF suggests that this molecule may be involved in the transcriptional regulation of genes ultimately controlling monocyte differentiation. In this regard, co-transfection experiments in which a LF expression vector was used together with a vector carrying a reporter gene linked to the GM-CSF promoter, revealed that LF reduces the activity of the GM-CSF promoter [45]. Consistent with these results, we found that bLF nuclear localization correlates with bLF capacity to stimulate IL-6 expression. The different capacity of monocytes to interact with bLF with respect to iMD-DCs may provide, at least in part, an explanation for the opposite effects, anti-inflammatory *versus* immunostimulatory [17], [18], observed in these cell types.

The mechanism of LF entry is still unknown, but is thought to occur via a LF receptor. LF is a cationic protein capable to bind, with different grade of specificity, a variety of cellular determinants, including bacterial components, strongly anionic molecules, CD14, and pathogen recognition receptors including C-type lectin receptors and TLR4 [1], [19], [21], [46]. Although the relevance of these receptors in triggering bLF effects in human primary monocytes and MD-DCs is unknown, it is of interest that major differences in the expression of at least some of these receptors (e.g. CD14 and DC-SIGN) have been reported in monocytes *versus* iMD-DCs. In particular, CD14 takes part in receptor complexes that play a crucial role in governing inflammation such as TLR4 [47] and TLR2 [48] pathways. In this regard, our study demonstrates that although not involved in bLF uptake by monocytes, CD14 and its co-receptors TLR2 and TLR4 play a role in bLF-induced signalling leading to IL-6 expression. Since CD14 is barely or not expressed at all in MD-DCs, these results suggest that this receptor may represent an important determinant for the differential effects induced by bLF in the two cell types.

Taken together these results shed some light on the mechanisms underlying bLF anti-inflammatory activity, highlighting the importance of monocytes as a preferential target for this molecule, and providing further support to its potential therapeutic application to inflammatory diseases.

## Materials and Methods

### Cell separation and culture

Peripheral blood mononuclear cells (PBMCs) were isolated from the peripheral blood of healthy donors by Ficoll-Paque density centrifugation [49]. Monocytes were further purified by depleting the non monocytic population by immunomagnetic bead selection as previously described [50]. Immature MD-DCs were generated by culturing monocytes in RPMI supplemented with 10% FBS containing GM-CSF and IL-4 (50 ng/ml and 500 U/ml, respectively, kindly provided by Schering-Plough, Dardilly, France) in the presence or in the absence of bLF (100 µg/ml) as previously described [51]. Freshly isolated monocytes were treated with bLF in the presence of differentiating factors soon after seeding, at day 2 and day 5 of culture, unless differently specified.

### Reagents

All culture reagents were purchased as endotoxin-free lots (Biowhittaker). LPS from *Escherichia Coli* (serotype EH100, Ra TLRgrade, Alexis Biochemicals), and poly(I-C) by Sigma. Resiquimod (R848) was a gift of Philippe Neuner (Istituto Ricerche di Biologia Molecolare, Pomezia, Italy). Highly purified bLF in lyophilized form was kindly provided by Morinaga Milk Industries Co., Ltd., (Tokyo, Japan). bLF was checked for purity [52], iron saturation and endotoxin content as previously described [52]. Monoclonal Abs (mAbs) against TLR4 [53] (5 µg/ml, clone 15C1) and TLR2 [54] (5 µg/ml, clone T 2.5) were kindly provided by Greg Elson. Monoclonal Abs against CD14 (5 µg/ml, clone 134620) and IgG1k isotype control Abs (5 µg/ml, clone 11711) were purchased from R&D. MD-DC phenotype was characterised by using the following mAbs: FITC-CD1a and PE-CD1a (clone HI149), FITC-CD14 and PE-CD14 (clone MΦP9), FITC-CD40 (clone 5C3), FITC-CD80 (clone L307.4), FITC-CD86 (clone 2331(FUN-1)), FITC-CD83 (clone HB15e), FITC-HLA-DR (clone G46-6), FITC-HLA-ABC (clone G46-2.6), FITC-ILT3 (clone 293623, R&D), FITC-ILT4 (clone 287219, R&D), PE-CD274 (PD-L1, clone MIH1), PE-CD206 MR (clone 19.2), mouse purified anti-CD209 (DC-SIGN, DCN46) followed by FITC-goat-anti-mouse IgG (H+L) F(ab')2 (PIERCE). Non specific binding was checked by the respective isotype Abs FITC-IgG2a (G155-178), FITC-IgG1 and PE-IgG1 (clone MOPC-21), FITC-IgG2a (clone 20102, R&D), purified mouse IgG2_b_k (clone 27–35). Unless differently indicated, mAbs were purchased from BD Biosciences. Briefly, 2–5×10^5^ cells were processed and labelled as previously described [55]. Samples were acquired with a FACS Calibur flow cytometer by using Cell Quest (Becton Dickinson) and data analyzed by FlowJo (Tree Star, Inc.).

### Antigen-uptake assay

MD-DCs were stimulated with LPS or poly(I-C) at day 6 of culture. 24 h later, cells were washed and incubated with FITC-labeled DXT (Molecular Probe, 500 µg/sample) for 40 min at 37°C, or 0°C to test unspecific binding. Cells were then fixed in PBS/4%PFA and analyzed by flow cytometry.

### DC-T cell cultures and Th profile

Allogenic CD4^+^ naïve T cells were isolated from PBMC of healthy donors by using naïve CD4^+^ T cell isolation Kit II (Miltenyi Biotech) and co-cultured in RPMI 5% human AB pool serum with MD-DCs or bLF-MD-DCs primed with LPS (10 ng/ml) or poly(I-C) (20 µg/ml), at DC-T ratio of 1∶10, 1×10^6^ T cells/well in 24-well plates. At day 5, supernatants were collected for cytokine determination while cells were extensively washed, re-suspended in fresh medium at 1×10^6^ cell/ml and stimulated with ionomycin (2 µg/ml) and PMA (50 ng/ml) (both from Sigma-Aldrich) for 5 h. Golgi Stop (BD Bioscience) was added during the last 3 h of culture following the manufacturer's instructions. After stimulation, CD4^+^ T cells were fixed, permeated using Cytofix/Cytoperm Plus™ (BD Bioscience) and processed for intracellular staining with Fastimmune FITC-IFN-γ/PE-IL-4 cocktail (clones 25723.11 and 3010.211 respectively, BD Bioscience), and PE-CD154 [56] (clone TRAP1, BD Bioscience). Cells were also stained with the respective isotype Abs mouse FITC-IgG2a/PE-IgG1 (clones ×39/×40, BD Biosciences) and mouse PE-IgG1 (MOPC-21, BD Biosciences). Alternatively, allogenic CD4^+^ naïve T cells were primed with LPS-activated MD-DCs or bLF-MD-DCs at T/DC ratio of 1∶10 for 7–10 days and successively re-stimulated with allogenic cryopreserved DCs of the same donor. Seven-to ten days after last stimulation, cells were processed for surface staining with FITC-CD25 (clone M-A251, BD Biosciences), then fixed and permeated for intracellular APC-FoxP3 (clone PCH101, eBioscience) staining and FACS analysis.

### T cell proliferation assay

MD-DCs were co-cultured at different DC/T cell ratio, starting from 1∶10, with peripheral blood lymphocytes (PBL), obtained from PBMCs after depletion of CD14^+^ cells. After 5–10 days, cells were fixed and permeated as above described, then stained with Ki67 and the relative isotype Ab, following the manufacturer's instructions (FITC Mouse Anti-Human Ki67 Set, BD Biosciences).

### Determination of cytokine and chemokine levels

Culture supernatants were processed by ELISA for the following cytokines: TNF, IL-12, IL-10 (sensitivity 32.5 pg/ml, homemade assay, Pierce Endogen), IL-6 (sensitivity 7.8 pg/ml, ELISA MAX™ Set, BioLegend), CCL2 (sensitivity 15.6 pg/ml, homemade assay, R&D System), IL-23 (sensitivity 31 pg/ml, eBioscience) and IL-2 (sensitivity 7.8 pg/ml, ELISA MAX™ Set, BioLegend).

### Cytoplasmic proteins expression analysis

Immunoblotting analysis of STAT3, SOCS-3 and IDO was carried out in cells differentiated for 6 days in the presence or in the absence of bLF. Endogenously produced IL-6 was neutralized by anti-human-IL-6 (5 µg/ml, clone 6708.11, SIGMA) or IgG1k isotype control (5 µg/ml, clone 11711, R&D) mAbs. Cells were pre-treated at day 0, 3 and 6 with anti-IL-6 for 30 min, before bLF addition to the cultures. Six h after the last bLF treatment whole cell proteins were extracted as previously described [57]. The protein concentration was determined using the Bio-Rad protein assay (Hercules, CA) according to the manufacturer's instructions. Twenty micrograms of lysate was separated by SDS-PAGE on a 10% gel and electroblotted to nitrocellulose filter (Protran BA 85, Schleicher & Schuell, Keene, NH). The following Abs were used: rabbit polyclonal Ab anti-phospho-STAT3 (Y705; Cell Signaling Technology, diluted 1∶1000), mouse mAb anti-STAT3 (BD Transduction Laboratories, diluted 1∶2500), anti-Actin Ab-5 (BD Biosciences, diluted 1∶5000), anti-SOCS-3 (Santa Cruz Biotechnology, diluted 1∶500) and anti-IDO (Upstate, diluted 1∶500). Signals were revealed after incubation with anti-mouse or anti-rabbit Ig HRP secondary Ab followed by ECL detection reagent (Amersham).

### Confocal laser-scanner microscopy analysis (CLSM)

Cells were fixed with 1% PFA for 15 min at room temperature (RT), and permeabilized with Dulbecco's PBS (DPBS), containing Ca^++^ and Mg^++^, 1% BSA and 0,1% Triton X-100 (Sigma-Aldrich Co.) for 30 min at RT. Cells were stained with a 1∶50 dilution in DPBS/0.1% BSA of FITC-conjugated polyclonal rabbit anti-human LF Ab (DakoCytomation) for 1 h at RT. As a negative control, the primary Ab was omitted. Cells were then extensively washed with DPBS and stained with the nuclear fluorescent probe TO-PRO-3 (1 mM, Molecular Probes) for 15 min at RT. After several rinses, cover slips were mounted in buffered glycerol (pH 9) and sealed with nail polish. Immunofluorescence imaging was performed using a Leica confocal microscope (Laser Scanning TCS SP2) equipped with Ar/ArKr and He/Ne lasers at ×40 magnification under an oil-immersion lens. A series of 12 optical sections with a step size of 1 µm through cells were acquired. Laser line was at 488 nm and 633 nm for FITC and TO-PRO-3 excitation, respectively. The percentage of positive cells was calculated by analyzing at least 350 cells for each experimental sample.

### Statistical analysis

Statistical comparison between different experimental conditions was determined by the Student's *t* test (paired, two-tailed) by using SPSS software. Differences were considered significant when *p* values were <0.05 (*), <0.01 (**), <0.001(***).

## Supporting Information

Figure S1 **bLF induced impairment of R848-induced MD-DC maturation.** Monocytes were stimulated to differentiate into iMD-DCs in the presence or in the absence of bLF as described in the legend to Figure 1. At day 5 of culture, cells were stimulated with R848 (2 µg/ml). (A) Twenty-four hours later, cells were collected and stained with a mAb to CD83. Open area represents staining with isotype Ab and shaded area CD83 expression in activated control and bLF-MD-DCs. Numbers indicate MFI. One representative experiment out of 3 is shown. (B–C) IL-12 and TNF contents in supernatants of MD-DCs stimulated with R848 for 24 h. Mean ± SE of 3 independent experiments is shown. ** *p*<0.01, MD-DCs *versus* bLF-MD-DCs.
(TIF)
