# Supplementary figures and images for: Bovine Lactoferrin Counteracts Toll-Like Receptor Mediated Activation Signals in Antigen Presenting Cells
Source: PLoS One. 2011 Jul 25;6(7):e22504. doi: 10.1371/journal.pone.0022504 (PMC3143167; doi:10.1371/journal.pone.0022504)

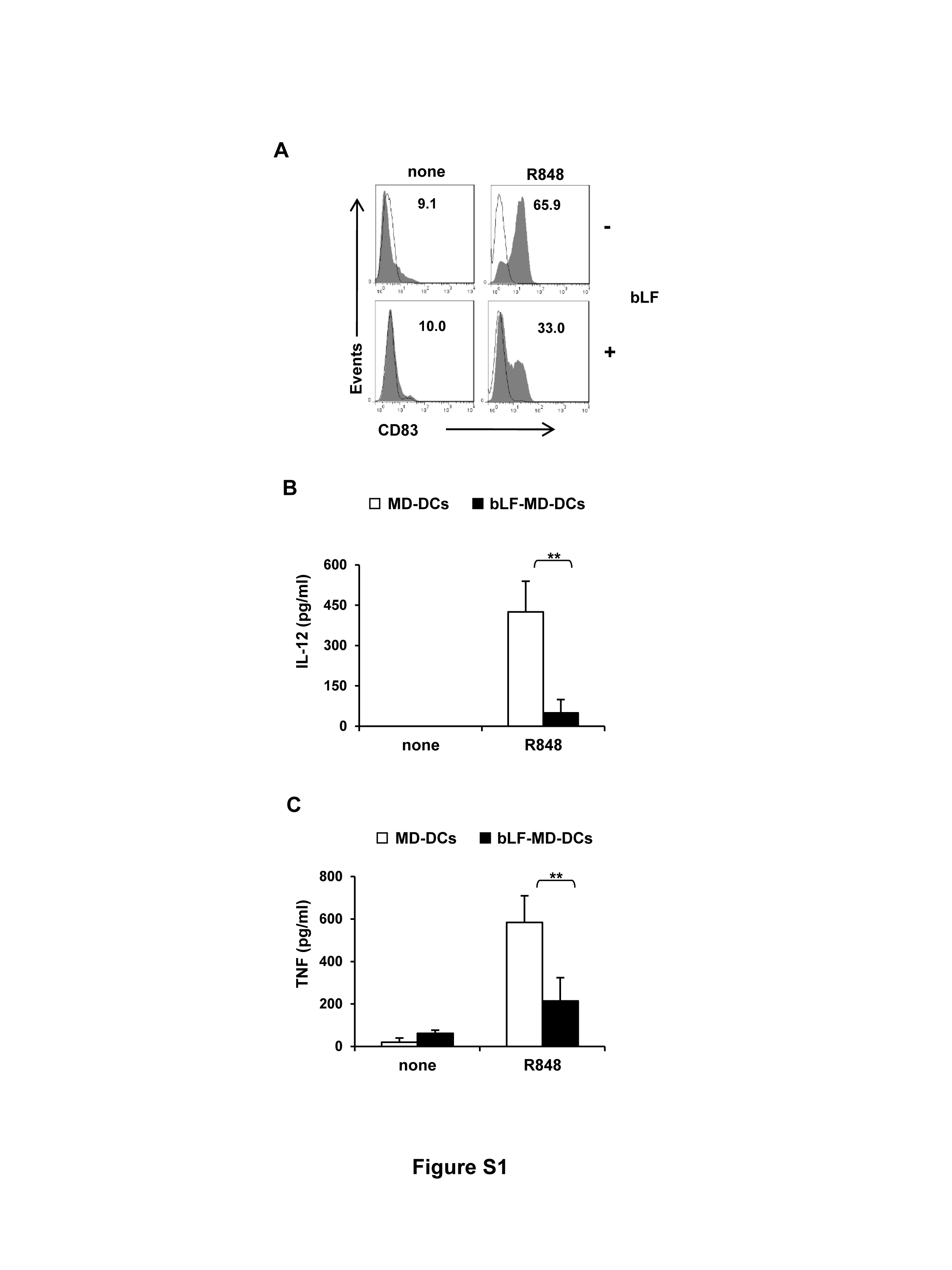

Supplement: Figure S1 — bLF induced impairment of R848-induced MD-DC maturation. Monocytes were stimulated to differentiate into iMD-DCs in the presence or in the absence of bLF as described in the legend to Figure 1. At day 5 of culture, cells were stimulated with R848 (2 µg/ml). (A) Twenty-four hours later, cells were collected and stained with a mAb to CD83. Open area represents staining with isotype Ab and shaded area CD83 expression in activated control and bLF-MD-DCs. Numbers indicate MFI. One representative experiment out of 3 is shown. (B–C) IL-12 and TNF contents in supernatants of MD-DCs stimulated with R848 for 24 h. Mean ± SE of 3 independent experiments is shown. ** p<0.01, MD-DCs versus bLF-MD-DCs. (TIF) [file pone.0022504.s001.tif]
